# Cross-cultural adaptation into Punjabi of the English version of the Hospital Anxiety and Depression Scale

**DOI:** 10.1186/1471-244X-7-5

**Published:** 2007-01-26

**Authors:** Deirdre A Lane, Jagdish Jajoo, Rod S Taylor, Gregory YH Lip, Kate Jolly, the Birmingham Rehabilitation Uptake Maximisation (BRUM) Steering Committee

**Affiliations:** 1 University Department of Medicine, City Hospital, Dudley Road, Birmingham B18 7QH, UK; 2 Department of Psychiatry, Dorothy Pattison Hospital, Walsall WS2 9XH, UK; 3 Department of Public Health and Epidemiology, University of Birmingham, Edgbaston, Birmingham B15 2TT, UK

## Abstract

**Background:**

We wanted to use a Punjabi version of the Hospital Anxiety and Depression Scale (HADS) to enable non-English speaking patients to participate in a clinical trial. The aim of the study was to translate and validate the Hospital Anxiety and Depression Scale into Punjabi.

**Methods:**

The HADS was translated into Punjabi by a multidisciplinary team, verified against the original version, and administered to 73 bilingual patients attending an outpatient clinic.

**Results:**

One sample t-tests and the Bland-Altman plots demonstrated acceptable linguistic agreement between the two versions of the HADS. Spearman's rank-order correlation coefficients (p < 0.0001) demonstrate excellent conceptual agreement between each item and its corresponding subscale score, for both versions. Concordance rates revealed that the Punjabi HADS adequately identified borderline cases of anxiety (80.8%), definite cases of anxiety (91.8%) and depression (91.8%), but was less reliable in identifying borderline cases of depression (65.8%). Cronbach alpha coefficients revealed high levels of internal consistency for both the Punjabi and English versions (0.81 and 0.86 for anxiety and 0.71 and 0.85 for depression, respectively).

**Conclusion:**

The Punjabi HADS is an acceptable, reliable and valid measure of anxiety and depression among physically ill Punjabi speaking people in the United Kingdom.

## Background

The Hospital Anxiety and Depression Scale (HADS) [1], specifically designed to identify possible anxiety and depressive symptoms in medical patients, has been used extensively as both a clinical and research tool. It has been demonstrated to be a reliable and valid instrument for assessing anxiety and depression in physically ill patients [2,3] and has been translated into many languages, including Arabic, Chinese, Dutch, French, German, Iranian, Italian, Spanish and Urdu.

Clinical and epidemiological trials often employ questionnaire measures to assess mood, but this requires all the participants to be able to read and/or speak the same language, which can be problematic. The current translation and validation of the HADS into Punjabi was undertaken to enable patients who did not speak fluent English to participate in the Birmingham Rehabilitation Uptake Maximisation (BRUM) study [4], a randomized controlled trial of home versus hospital-based cardiac rehabilitation, and to be assessed using instruments in their own language. Since approximately 20% of the target population of west Birmingham were of South Asian origin, and predominantly Punjabi speaking, it was deemed appropriate to translate the HADS into Punjabi, to prevent excluding participants merely on the basis of being non-English speaking.

The question of whether directly translated questionnaires encapsulate the same meaning as the original has been widely debated [5-7]. However, acceptable linguistic and conceptual equivalence has been demonstrated in the translation of the English version of the HADS into another South-Asian language, Urdu [8], where it has also been shown to be reliable and valid measure of depression and anxiety [8].

The aim of the present study was to translate and validate the Hospital Anxiety and Depression Scale (HADS) into Punjabi.

## Methods

### Translation

Translation of the HAD scale [1] was discussed among a trans-cultural, multidisciplinary team consisting of doctors, a nurse, interpreter/translator, and Punjabi and English speaking lay members. The team was instructed to produce a conceptual translation of the HADS. There was considerable discussion both about the equivalent meanings of each of the items, as well as the gradations of frequency (e.g., "lot of the time", "a great deal of the time"). The English version of the HADS was translated into the Punjabi language. Once the translation had been completed and approved by the team, two Indian Punjabi-speaking doctors and a Punjabi-speaking nurse independently checked the first translation against the English version. No changes were suggested. The approved Punjabi version was then made available in written form, on audiocassettes and CDs.

### Hospital Anxiety and Depression Scale questionnaire

The HADS (*see Appendix for copyright statement*) consists of 14 items to assess anxiety (7 items) and depression (7 items). Each item is rated from 0 to 3, with higher scores indicating greater anxiety or depression. The maximum score on either subscale is 21, with scores of 8 to 10 representative of 'borderline' psychological morbidity and scores of 11 or more indicative of a significant 'case' of depression or anxiety.

### Participants

Participants were eligible to take part in this study if they spoke and read both English and Punjabi. Ninety people of South-Asian origin, who described themselves as fluent in both English and Punjabi, attending the hypertension, cardiology, and diabetic clinics at the City Hospital, Birmingham, between November 2001 and July 2002 were approached to participate. Of these, 81 (90%) agreed to join the study, with 73 (90.1%) providing complete data for both versions of the HADS. All participants completed the HADS questionnaires in both English and Punjabi, the administration of which was counterbalanced to prevent order effects. A demographic questionnaire was administered between the two versions of the HADS to reduce contamination of completion of the second HADS by the first. In order to assess the internal consistency between the English and Punjabi versions of the HADS they were administered on the same day rather than a week or two apart, given that the HADS assesses current feelings of anxiety and depression.

### Data analysis

Linguistic equivalence, the extent to which the translation is a literal one, was assessed by calculating the mean difference for each item (Punjabi minus English) and analysed using one-sample t-tests. Conceptual equivalence, the degree to which the Punjabi version captures the meaning of the original HADS, was assessed by Spearman's rank-order correlation coefficients for each item and its corresponding subscale score and within each item (on the two versions). A correlation of 0.3 or more indicates that a particular item is consistent with the content of the overall scale [9]. To illustrate the agreement between the English and Punjabi sub-scores for anxiety and depression, a mean difference (and 95% confidence interval) was calculated and a Bland-Altman plot (plot of individual differences against the mean) was produced [10]. Scale equivalence, the extent to which the Punjabi and English versions of the HADS identify the same individuals as either a definite or borderline case of anxiety and/or depression, was determined by simultaneously classifying each participant as either anxious or depressed on the two versions, and calculating the concordance rate between them. To test reliability, the internal consistency of the Punjabi and English HADS was assessed using Cronbach's alpha coefficient (α), with α ≥ 0.70 considered adequate [9]. It has been recommended that α should be at least 0.60 for a self-report instrument to be reliable and at least 0.80 when used as a screening tool [9]. All tests were two-tailed, with p-values ≤ 0.05 considered statistically significant. Data was analysed using SPSS for Windows, Version 12.0.

## Results

The mean (SD) age of the 73 participants was 47.2 (14.2) years, and 63.0% were male. Fifty-four (76.1%) described their ethnic group as Indian, 15 (21.1%) as Pakistani, and four described themselves as 'Other'.

### Linguistic equivalence

Table 1 presents the mean (SD) scores and the mean (SD) difference scores (Punjabi minus English) for the each of the 14 items of the HADS. One sample t-tests demonstrated that five items, A3, A11, D2, D12, and D14, had statistically significant differences, with the Punjabi scores being significantly higher for three items (A11, D2, and D14). Mean differences of ± 0.25, in the context of a Likert scale scored from 0 to 3, are considered acceptable. Only four items, A3, A11, D2, and D12, had mean differences of greater than ± 0.25. The mean (SD) anxiety subscale score on the Punjabi and English version of the HADS was 8.36 (4.79) and 8.16 (4.88), respectively. The analogous figures for depression were 7.04 (3.97) and 6.52 (4.67), respectively. Examining differences at the scale level, demonstrated that the subscale scores of the Punjabi and English versions of the anxiety (t = 0.60, df = 72, p = 0.55) and depression (t = 1.36, df = 72, p = 0.18), and the overall distress (t = 1.28, df = 72, p = 0.21) scores were not significantly different.

**Table 1:** Mean (SD) scores and mean difference scores for each item and total scores on the Punjabi and English version of the HADS†

**Item**	Mean (SD) score on Punjabi version	Mean (SD) score on English version	Mean (SD) difference in scores†
**Anxiety**			
A1 (Tenseness)	1.11 (0.97)	1.10 (0.87)	0.01 (0.83)
A3 (Frightened)	1.07 (1.07)	1.40 (1.02)	-0.33 (1.06)*
A5 (Worry)	1.14 (1.08)	1.16 (0.97)	-0.03 (1.00)
A7 (Relaxed)	1.14 (1.03)	1.01 (0.95)	0.12 (0.88)
A9 (Nervousness)	0.93 (0.89)	0.89 (0.91)	0.04 (0.84)
A11 (Restlessness)	1.63 (1.03)	1.37 (0.92)	0.26 (1.09)*
A13 (Panic)	1.34 (0.99)	1.23 (1.03)	0.11 (0.89)
Anxiety subscale score	8.36 (4.79)	8.16 (4.88)	0.19 (2.71)
			
**Depression**			
D2 (Current enjoyment)	1.25 (1.02)	0.96 (0.90)	0.29 (1.11)*
D4 (Laughter)	0.86 (1.00)	0.71 (0.99)	0.15 (0.95)
D6 (Cheerfulness)	0.85 (0.70)	0.82 (0.86)	0.03 (1.09)
D8 (Reduced energy)	1.40 (0.94)	1.41 (0.90)	-0.01 (1.07)
D10 (Loss of interest)	1.07 (1.10)	0.90 (0.97)	0.16 (0.91)
D12 (Future enjoyment)	0.77 (0.87)	1.05 (1.03)	-0.29 (1.03)*
D14 (Leisure time enjoyment)	0.85 (0.94)	0.66 (0.89)	0.19 (0.74)*
Depression subscale score	7.04 (3.97)	6.52 (4.67)	0.52 (3.28)
			
Total HADS score	15.40 (8.24)	14.68 (8.61)	0.71 (4.76)

† = Punjabi score minus English score

* = p < 0.05 (based on one sample t-tests)

**Figure 1:**
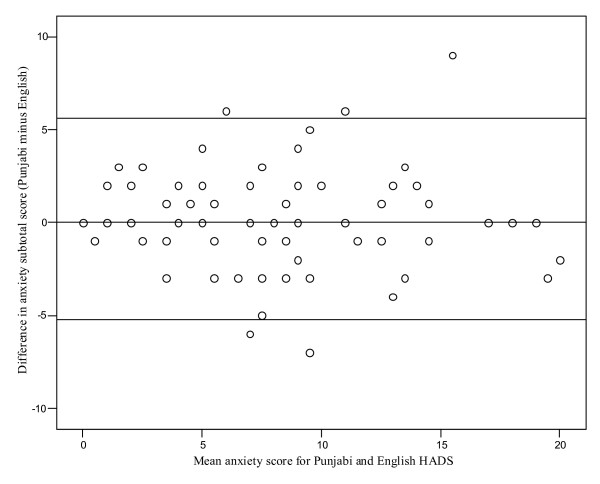
**Bland-Altman plot for the anxiety subscale for Punjabi and English versions of the HADS**. Mean difference ± 2 standard deviation cut-offs shown.

**Figure 2:**
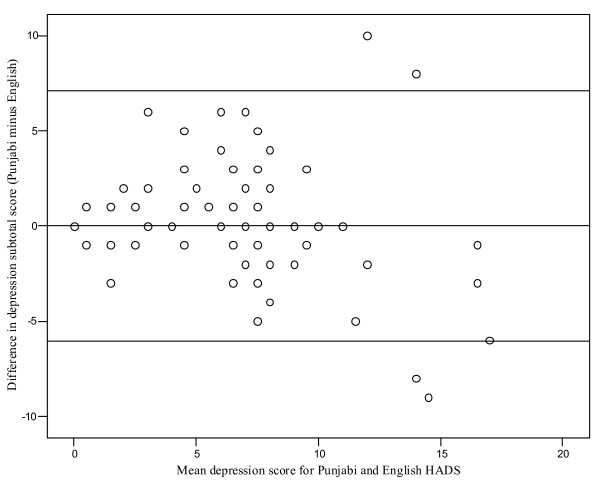
**Bland-Altman plot for the depression subscale for Punjabi and English versions of the HADS**. Mean difference ± 2 standard deviation cut-offs shown.

The Bland-Altman plots (Figures 1 and 2) depict the degree of agreement between the Punjabi and English anxiety and depression sub-scale scores. The difference in the mean sub-scale scores (Punjabi minus English) was plotted against the mean subscale score for both versions. The mean (95% CI) difference for the anxiety and depression sub-scale scores was 0.19 (-5.23 to 5.61) and 0.52 (-6.04 to 7.08), respectively. There does not appear to a significant difference between Punjabi and English scores for either depression or anxiety. Overall, 93.2% of points lay within the 95% confidence intervals for the anxiety sub-scale, and 94.5% for the depression sub-scale.

### Conceptual equivalence

Spearman rank-order correlation coefficients for each item and its' corresponding subscale score ranged from 0.58 to 0.80 and 0.64 to 0.83 for the anxiety subscale scores for Punjabi and English versions of the HADS, respectively. The analogous figures for depression were 0.41 to 0.67 and 0.46 to 0.77, respectively [see Table 2]. All correlation coefficients were highly significant (p < 0.0001). For all items, with one exception (A13- Related to panic), the p-value for the difference between the pair of correlation coefficients was ≥ 0.05. For half of the items, this difference exceeded 0.10.

**Table 2:** Spearman's rank-order correlation coefficients for each item on the Punjabi and English versions of the HADS with its corresponding subscale score

Item	Spearman's *rho *for Punjabi version of HADS	Spearman's *rho *for English version of HADS
**Anxiety**		
A1 (Tenseness)	0.80**	0.70**
A3 (Frightened)	0.73**	0.79**
A5 (Worry)	0.60**	0.83**
A7 (Relaxed)	0.58**	0.73**
A9 (Nervousness)	0.58**	0.49**
A11 (Restlessness)	0.58**	0.64**
A13 (Panic)	0.77**	0.81**
		
**Depression**		
D2 (Current enjoyment)	0.64**	0.76**
D4 (Laughter)	0.67**	0.73**
D6 (Cheerfulness)	0.41**	0.77**
D8 (Reduced energy)	0.57**	0.46**
D10 (Loss of interest in self)	0.52**	0.66**
D12 (Future enjoyment)	0.51**	0.73**
D14 (Leisure time enjoyment)	0.67**	0.59**

** p < 0.0001

### Scale equivalence

Each participant was classified as being a borderline (scores of ≥ 8) or definite (scores of ≥ 11) case of anxiety or depression on both the Punjabi and English versions of the HADS. Table 3 shows that the concordance rates for borderline cases were 80.8% and 65.8% for anxiety and depression, respectively. The analogous figures for definite cases were 91.8% for both anxiety and depression. The corresponding kappa coefficients for borderline anxiety and depression and definite anxiety and depression were 0.61 and 0.30, and 0.80 and 0.62, respectively. The findings indicate that both the Punjabi and English versions of the HADS were more than adequate in declaring people to be borderline cases of anxiety, and definite cases of anxiety or depression. However, they were less reliable in identifying borderline cases of depression.

**Table 3:** Anxiety and depression 'cases' (borderline and definite) according to the Punjabi and English versions of the HADS

	**English HADS**			
	Borderline cases (scores ≥ 8)		Definite cases (scores ≥ 11)	
**Punjabi HADS**	Non-case	Case	Non-case	Case
**Anxiety**				
Non-case	26	8	49	2
Case	6	33	4	18
Concordance (%)	80.8		91.8	
Kappa coefficient	0.613		0.80	
				
**Depression**				
Non-case	32	7	61	3
Case	18	16	3	6
Concordance (%)	65.8		91.8	
Kappa coefficient	0.297		0.620	

### Reliability

The Cronbach's alpha coefficient for anxiety on the English and Punjabi versions was 0.86 and 0.81, respectively. The analogous figures for depression were 0.85 and 0.71, respectively. These findings indicate high internal consistency for both anxiety and depression on both the Punjabi and English versions of the HADS.

## Discussion

This study demonstrates that the Punjabi translation of the HADS had reasonable linguistic equivalence, i.e., the translation from English into Punjabi was a literal one, with only five of the 14 items displaying significant mean difference scores. The rewording of these five questions may have attenuated the variation between the two versions. These differences may be due to the fact that the translation was trying to encapsulate the original meaning of the English version rather than producing a literal translation. The Urdu translation of the HADS [8] demonstrated that the Urdu scores were generally higher than the English scores for the same item but in no instance did the mean difference between the scores exceed ± 0.25. The different sample population employed for the two validation studies may also explain these differences: second year medical students in Lahore [8] versus medical outpatients in Birmingham in the present study.

The tests of conceptual equivalence demonstrated that the Punjabi and English versions of the HADS were comparable for each item with its subscale score, with the exception of D6 (Related to cheerfulness). Each item on both the Punjabi and English versions correlated significantly (p < 0.0001) with its corresponding subscale score. For half of the items (A5, A7, D2, D6, D8, D10, and D12), the differences between the correlation coefficients were greater than 0.10, indicating less conceptual equivalence. In the Urdu validation study [8], most items correlated similarly with their respective subscale score in both English and Urdu. The correlation coefficients for items on the Punjabi and English anxiety subscales were slightly higher in the present study (0.58 to 0.80 and 0.64 to 0.83, respectively) compared to the corresponding Urdu scores (0.51 to 0.74 and 0.48 to 0.73, respectively) [8]. Correlation coefficients were similar for English depression scores (0.46 to 0.77) and lower for Punjabi depression scores (0.41 to 0.67) in the present study, when compared to the Urdu validation (0.52 to 0.69 and 0.58 to 0.71, respectively).

The evaluation of scale equivalence demonstrated that there was no significant difference in the mean anxiety or depression subscale scores between the two versions. In addition, the Punjabi version of the HADS did not display any tendency to over- or under-identify definite cases of anxiety or depression. Similarly, the Punjabi anxiety subscale adequately identified borderline cases of anxiety and those who were not anxious, demonstrating an 80.8% concordance rate, with a kappa coefficient of 0.61. However, the Punjabi depression subscale did tend to overestimate the number of borderline depressed participants, with a corresponding low kappa coefficient (0.297). These concordance rates are similar to those found in the validation of the Urdu HADS [8] for anxiety (borderline and definite cases) and definite cases of depression but the borderline depression concordance rate was much lower in the present study (87.0% vs. 65.8%) [8].

Tests of the reliability of the Punjabi version of the HADS reported Cronbach's alpha coefficients of 0.81 and 0.71 for the anxiety and depression subscales, respectively. The analogous figures for the English version were 0.86 and 0.85, respectively. These figures are comparable with internal consistency findings from the validation of the Iranian, Spanish, and Finnish HADS [11-13], particularly the anxiety subscale (0.78, 0.85, and 0.83, respectively), suggesting that the Punjabi version of the HADS has acceptable internal consistency and is a reliable and valid self-report instrument [9].

A common criticism is that although the original English version of the HADS has been translated into many other languages, often the new and original version are not administered to the same people who are bilingual and the results compared. It is therefore, not known whether these versions are equivalent to the English version, or if their scores are influenced by cultural factors [2]. However, there is evidence from the present study and the Urdu HADS translation [8], to suggest that both scales are comparable when completed by people who are bilingual. Cultural differences in the expression and reporting of psychological symptoms and emotions are paramount among South-Asians, who tend to somatise such symptoms. Therefore, it is important to have such tools available in different languages that have been shown to be comparable with the English version, to enable studies to assess symptoms of anxiety and depression in different ethnic/cultural groups.

Further, a recent review [14] of studies examining the psychometric properties of the HADS suggests that it has a tri-dimensional rather than a two-dimensional structure, comprising depression (or anhedonia), with the anxiety component divided into negative affectivity and autonomic arousal. Therefore, the physiological aspects of disease may influence patient's responses to the somatic items, which will fluctuate with changes in health status and certainly impact on the accuracy of case detection. The present study is limited by its analysis of the two original factors, anxiety and depression, and therefore, further research should examine this tri-dimensional structure, especially in medically-ill patients, where the physical aspects of the disease may be a more prominent driving force.

### Limitations

The present study has a number of limitations. First, a formal backward-forward translation was not conducted. However, three bilingual health professionals, not involved in the translation process, independently assessed equivalence of the Punjabi items with the original English version. Although this is not the 'gold standard' method for translation, this technique has been employed previously in translation of the HADS. Second, rewording of the five items which demonstrated a significant difference between the Punjabi and English version may have improved the literal translation of these items. However, our aim was to encapsulate the original meaning rather than producing an exact translation. Third, the Punjabi version was validated in a relatively small sample population compared to other contemporary studies examining factor analysis of the HADS. However, the present sample size was not that dissimilar to other questionnaire validation studies in South-Asian languages [8,15]. Finally, although this study demonstrated the accuracy of the Punjabi and English versions to detect 'cases' and borderline anxiety cases, caution is warranted in their ability to detect borderline cases of depression.

## Conclusion

This study has shown that the Punjabi version of the HADS is a reliable and valid measure of depression and anxiety in medical patients. The Punjabi version of the HADS enables Punjabi-speaking patients who are not fluent in English to take part in clinical trials that use the HADS as an outcome measure.

## Competing interests

The author(s) declare that they have no competing interests.

## Authors' contributions

DL undertook the statistical analysis and drafted the paper. JJ assisted with the translation, collected data, and undertook some analysis. RT provided statistical advice. GYHL helped to design the study. KJ conceived of the study and its design and collected some data. All the authors read and approved the final manuscript.

## Appendix

HADS copyright ^© ^R.P. Snaith and A.S. Zigmond, 1983, 1992, 1994. Record forms originally published in Acta Psychiatrica Scandinavica, 67, 361-70, copyright ^© ^Munksgaard International Publishers Ltd, Copenhagen 1983. Reproduced by permission of the Publishers, nferNelson Publishing Company Ltd., of The Chiswick Centre, 414 Chiswick High Road, London W4 5TF UK. All rights reserved including translation. nferNelson is a division of Granada Learning Limited, part of ITV plc.

## Pre-publication history

The pre-publication history for this paper can be accessed here:


